# A U-shaped association between dietary inflammatory index and oral pain: a cross-sectional study from NHANES 2003–2018

**DOI:** 10.3389/fnut.2025.1535241

**Published:** 2025-07-28

**Authors:** Honglan Sun, Chao Yang, Shizhao Chen, Xiaoyunqing Yin, Yuqi Huang, Huifang Kuang, Wen Luo

**Affiliations:** ^1^The First Clinical College/First Affiliated Hospital/School of Stomatology, Department of Stomatology, Key Laboratory of Emergency and Trauma of Ministry of Education, Key Laboratory of Hainan Trauma and Disaster Rescue, Hainan Medical University, Haikou, Hainan, China; ^2^Research and Development Department, Chengdu Shiliankangjian Biotechnology Co., Ltd., Chengdu, Sichuan, China

**Keywords:** dietary inflammatory index, oral pain, cross-sectional study, NHANES, U-shaped association

## Abstract

**Objectives:**

Oral pain (OP) is a prevalent condition affecting one in four US adults, potentially influenced by diet through inflammation-related pathways. This study aimed to investigate the association between the Dietary Inflammatory Index (DII) and the prevalence of OP in a nationally representative sample.

**Methods:**

This cross-sectional study included 23,869 participants from the 2003–2018 National Health and Nutrition Examination Survey (NHANES). OP was assessed via a self-reported question regarding the experience of OP in the past year. DII scores were calculated using data from two 24-h dietary recall interviews. Weighted multivariable logistic regression models and subgroup analyses were performed to evaluate the relationship between DII and OP. Restricted cubic spline analysis was used to examine the shape of the association.

**Results:**

DII was positively associated with OP in the fully adjusted regression model (OR: 1.026, 95% CI: 1.001, 1.051). The association between DII and OP presented a U-shape, with a turning point of 0.95, indicating that both low and high levels of dietary inflammation were associated with an increased risk of OP. Subgroup analyses revealed no significant differences across different stratifications (*p* > 0.05 for all).

**Conclusion:**

A U-shaped association between dietary inflammatory potential and oral pain was identified in this nationally representative sample. Encouraging a balanced diet that avoids both pro-inflammatory and excessively anti-inflammatory extremes may serve as a preventive or therapeutic strategy to alleviate oral pain and improve overall oral health outcomes.

## Introduction

Oral diseases, mainly encompassing dental caries and periodontal disease, are a significant public health concern affecting over 3.5 billion people worldwide (1). Oral pain (OP) is one of the most common and debilitating symptoms of oral diseases, characterized by an unpleasant sensory and emotional experience associated with actual or potential tissue damage (2). A study in the US based on the 2015–2018 National Health and Nutrition and Examination Survey (NHANES) showed that one out of four adults had OP, and 4% reported oral health-related productivity loss in the past year (3). OP is associated with a range of adverse psychosocial outcomes, including increased depression and anxiety and decreased quality of life (4). In addition, OP is a leading cause of reduced productivity, with the odds for oral health-related productivity loss being 13.85 times higher among individuals with OP (3). Furthermore, OP imposes a significant burden on the healthcare system, leading to increased medical costs related to consultation and treatment (5).

The etiology of OP is multifactorial, encompassing a variety of genetic, physiological, psychological, and environmental factors (6, 7). Among these factors, inflammation has emerged as a critical underlying mechanism in the development and persistence of OP (8, 9). Evidence suggests that oxidative stress can induce inflammasome activation, leading to subsequent release of inflammatory cytokines such as interleukin-6 (IL-6), C-reactive protein (CRP), and tumor necrosis factor-alpha (TNF-*α*), which play a crucial role in the exacerbation of neuropathic OP^7^. Increasing evidence also suggests that diet plays a pivotal role in modulating systemic inflammation and thus is a potential modifiable risk factor of OP (10, 11). Studies show that certain dietary constituents that contain polyphenol compounds, carotenoids, and fatty acids may attenuate noxious sensory information by relieving nociceptive and/or pathological pain (10). Araújo et al. (11) conducted a randomized controlled trial (RCT) to test the effectiveness of a four-week gluten-free diet intervention among women with myofascial pain. Compared to the control group, the experiment group showed a significant reduction in pain intensity and an increase in pressure pain threshold (PPT), indicating the potential of diet therapy as an important alternative treatment plan for OP (11).

To improve the specificity of dietary scores, Shivappa et al. (12) developed the dietary inflammatory index (DII) to evaluate an individual’s dietary inflammatory potential according to the pro- and anti-inflammatory properties of various dietary components. DII is a comprehensive tool for diet quality and estimates the inflammation effects of dietary consumption of 45 nutrients associated with six inflammatory biomarkers: IL-1β, IL-4, IL-6, IL-10, TNF-*α*, and CRP (13, 14). DII is widely used to explore the relationship between diet and disease outcomes mediated through inflammation-related pathways (12). Previous studies reported that higher DII was associated with increased risks of metabolic disease (15), kidney disease (16), dementia (17), joint pain (18), headache (19), and mortality of all non-communicable diseases (20). Furthermore, accumulating evidence has also demonstrated positive associations between DII and several oral diseases, including oral cancer (21), periodontitis (22, 23), and gingivitis (24). These findings emphasize the essential role that DII plays in disease treatment and prevention.

However, little is known about the association between DII and OP in adults. To address the research gap, we conducted a cross-sectional study to explore the association between DII and OP based on a national sample from the NHANES database from 2003 to 2018. The findings may contribute to a deeper understanding of how diet-related inflammation correlates with OP, potentially guiding future longitudinal research that could inform dietary recommendations for OP management.

## Methods

### Data source and study population

The study extracted data from the NHANES for the years 2003–2018 for a cross-sectional analysis. NHANES is a nationwide survey to assess the health of the US population utilizing a multistage sampling method (25). The dataset includes sociodemographic information, physical examination results, and biological results.

**Figure 1:**
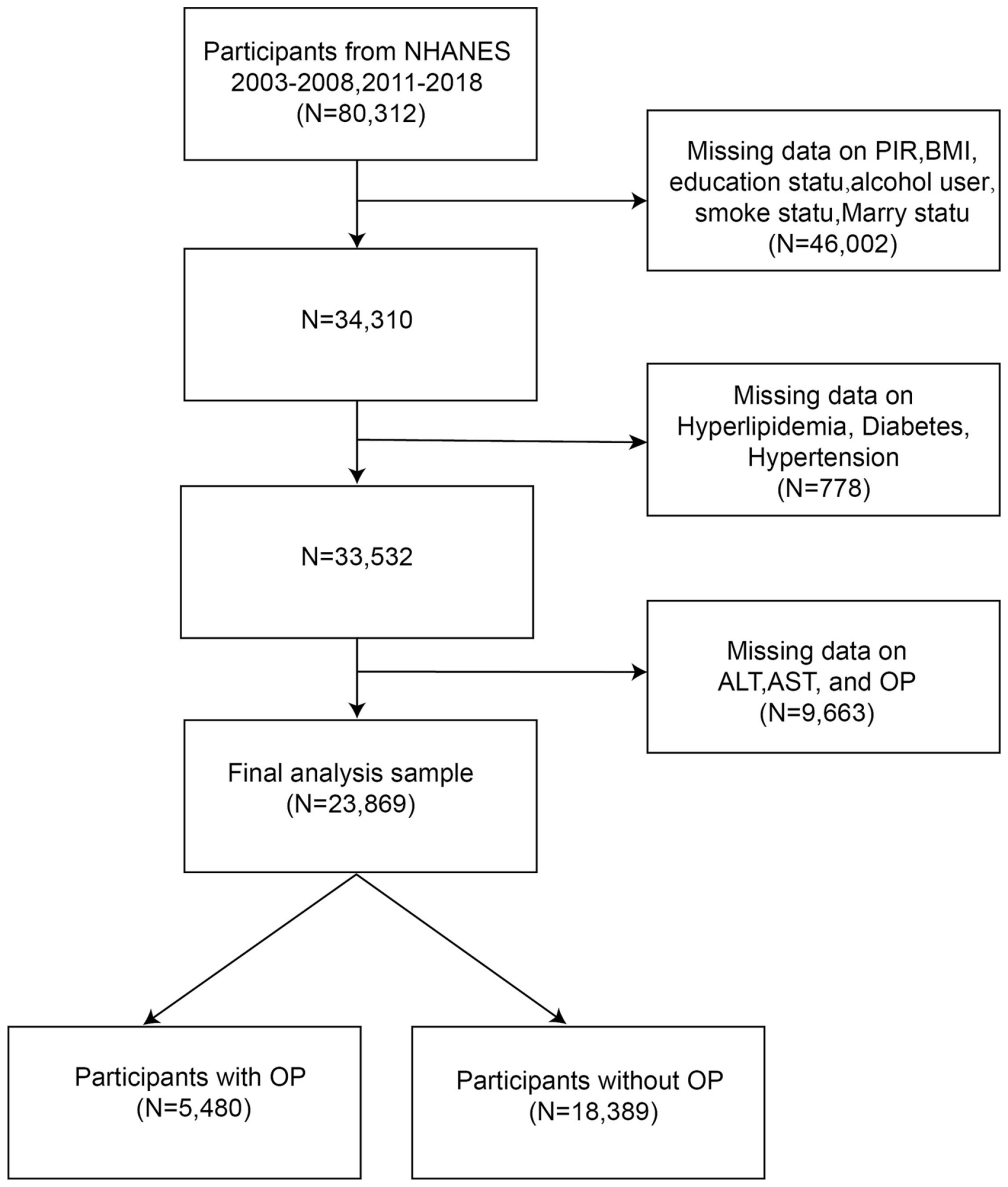
Flow chart of participants selection, NHANES.

Among 80,312 participants with complete information on DII, 56,443 were excluded due to missing data on critical variables. This led to a final sample size of 23,869, including 5,480 participants with OP and 18,389 participants without OP (Figure 1). Ethical approval was obtained from the National Center for Health Statistics Research Ethics Review Committee, and written informed consent was obtained from all participants.

### OP

OP was assessed based on one question in the NHANES: “Have you ever felt pain in the mouth in the past year?” with five response frequency options ranging from 0 (never) to 4 (very often). A cutoff of 2 was used to categorize the participants into the OP group (≥2) and the non-OP group (<2).

### Dietary inflammatory index (DII)

The DII was used to evaluate the dietary inflammatory function of food (12). It initially includes 45 food nutrients related to six inflammatory biomarkers: IL-1β, IL-4, IL-6, IL-10, TNF-*α*, and CRP (13, 14). In NHANES, DII was calculated based on 27 dietary components, which was comparable to the complete list of 45 in the prediction of inflammatory function (26). Due to the available data in the NHANES database, missing dietary component data were not imputed, and only the available data were used in the DII calculation to maintain the integrity and robustness of the results. Dietary intake was assessed by two interviews conducted 3 to 10 days apart to recall diet in the past 24 h.

The DII is calculated based on the global daily mean intake and standard deviation for each food derived from a global database. A Z-score is obtained by the following formula: (actual intake - global daily mean intake)/ standard deviation, which is then transformed into a percentile score to adjust for potential skewing. The global reference values used for calculating the DII were obtained from an extensive global database, as derived from Shivappa et al. (12). The overall DII score is obtained by summing up the percentile score multiplied by its specific inflammatory effect score for each food. Previous studies show that DII scores based on 25–30 food components range between − 5.5 and + 5.5 (27). A higher DII score suggests a more pro-inflammatory diet. We categorized the DII score into quartiles, defined as Q1(−5.103, 0.255), Q2(0.255, 1.794), Q3(1.794, 2.988) and Q4(2.988, 5.794), with Q1 as the reference group. DII was calculated using the following equation:

$\begin{array}{l} \mathrm{DII}\;(\text{for each component})=\\ \;\;\;\;\;\;\;\;\;\;\;\;\;\;\;\;Z\;{\text{score}}^{’}\times \text{Overall inflammatory effect score} \end{array}$

$\begin{array}{l} Z\;\text{score}=(\text{daily mean intake}-\text{global daily mean intake})\\ /\text{standard deviation} \end{array}$

$Z\;{\text{score}}^{’}=(Z\;\text{score}\to \text{converted to}\;a\;\text{percentile score})\times 2–1$

TotalDIIscore=∑(DIIfor each food component)

The value of global daily mean intake (units/d), standard deviation and overall inflammatory effect score were listed in Supplementary Table S2. The conversion of the Z-score to a percentile score is intended to mitigate the impact of right skewness, ensuring a symmetric distribution centered around 0, with values ranging from −1 (maximum anti-inflammatory effect) to +1 (maximum pro-inflammatory effect).

### Other covariates

We collected self-reported sociodemographic characteristics including age, sex, race or ethnicity, education level, and marital status. Smoking status was defined as never (< 100 cigarettes in life, and not currently smoking), former (> 100 cigarettes in life, and not currently smoking), and current smokers (> 100 cigarettes in life, and smoke some day or every day) (28). Body mass index (BMI) was calculated as weight in kilograms divided by height in square meters. Alcohol use was defined as never (<12 drinks in lifetime), former (> 12 drinks in lifetime, and not drinking last year), mild (females: ≥ 1 drink/ day, males: ≥ 2 drinks/ day), moderate (females: ≥2 drinks/day; males: ≥3 drinks/day, or binge drinking for 2–5 days/ month), heavy (females: ≥3 drinks/day; males: ≥4 drinks/ day, or binge drinking ≥ 5 days/month). Hypertension is defined by the following criteria: (1) three consecutive systolic blood pressure > 140 mmHg or diastolic blood pressure > 90 mmHg, (2) being told by a physician to have hypertension, or (3) having taken antihypertensive medications. Hyperlipidemia is defined by the following criteria: (1) TG ≥ 150 mg/dL, (2) TC ≥ 200 mg/dL, LDL-C ≥ 130 mg/dL, HDL-C < 40 mg/dL for males and <50 mg/dL for females, or (3) having used lipid-lowering medications. Diabetes mellitus (DM) is defined by the following criteria: (1) Being told by a doctor to have diabetes, or (2) having used anti-diabetic medications.

### Statistical analysis

Sample characteristics based on DII quartiles were compared using weighted chi-square tests for categorical variables and weighted t-tests for continuous variables. Based on the Variance Inflation Factor (VIF) analysis, all variables had VIF values ranging from 1 to 3, indicating minimal multicollinearity and confirming that the covariates included in the model are not highly correlated. Weighted multivariate logistic regressions were conducted to explore the association between binary oucome (OP) and multiple predictors (DII) in three different models, followed by a trend test to check their linear correlations. The weighting procedure was calculated as 1/7*wtmec2yr and represented the target population of 151,755,227 in the US. We used three models to analyze the association between DII and OP. Model 1 did not adjust for any potential confounders. Model 2 adjusted for age and sex. Model 3 was further adjusted for race, alcohol use, AST, ALT, BMI, smoke, hyperlipidemia, hypertension, and DM. Subgroup analyses were performed to examine whether the association between OP and DII was consistent across different sample characteristics such as age, gender, ethnicity, BMI, hypertension, and hyperlipidemia. Restricted cubic spline (RCS) was utilized to verify the nonlinear association between DII and OP. Weighted multivariable logistic regression was conducted using the *survey* package in R (version 4.2.2), and RCS analysis was performed using the *rms* package.

## Results

### Comparison of sample characteristics by OP status and DII quartile

Among the 23,869 participants included in the analyses, 5,480 participants had OP, with a prevalence rate of 22.96%. Table 1 shows the comparison of participants’ clinical and sociodemographic characteristics by OP. The OP group had a significantly higher DII score than the non-OP group (1.56 ± 0.04 vs. 1.35 ± 0.03, *p* < 0.001). Significant differences were also observed in all covariates except for hypertension, AST, and ALT. Supplementary Table S1 shows the comparison of participants’ clinical and sociodemographic characteristics by DII quartile. Participants consuming diets with greater inflammatory potential were more likely to have OP. They were predominantly women, less educated, and were more likely to have at least one of the following conditions: hyperlipidemia, diabetes mellitus, alcohol use, or smoking.

**Table 1:** Participants’ baseline clinical and sociodemographic characteristics by incidence of oral pain.

Variables	Total	Non-OP	OP	*p*
DII	1.40 ± 0.03	1.35 ± 0.03	1.56 ± 0.04	**< 0.001**
AST	25.46 ± 0.12	25.38 ± 0.14	25.77 ± 0.28	0.2
ALT	25.64 ± 0.15	25.50 ± 0.16	26.17 ± 0.35	0.08
Age	50.02 ± 0.23	50.71 ± 0.23	47.43 ± 0.31	**< 0.001**
Age group				**< 0.001**
20–39	28.47 (0.01)	27.38 (0.56)	32.55 (0.97)	
40–59	43.25 (0.01)	42.26 (0.56)	46.95 (0.99)	
> 60	28.28 (0.01)	30.36 (0.64)	20.50 (0.72)	
Sex				**0.005**
Female	50.95 (0.01)	50.33 (0.39)	53.27 (0.87)	
Male	49.05 (0.01)	49.67 (0.39)	46.73 (0.87)	
Race				**< 0.001**
Mexican American	7.43 (0.01)	7.12 (0.59)	8.55 (0.75)	
Non-Hispanic Black	9.90 (0.01)	8.98 (0.62)	13.36 (0.90)	
Non-Hispanic White	71.87 (0.03)	73.23 (1.19)	66.75 (1.53)	
Other Race	10.80 (0.00)	10.66 (0.50)	11.33 (0.73)	
Marry				**< 0.001**
Divorced	11.60 (0.00)	10.80 (0.33)	14.60 (0.64)	
Living with partner	6.74 (0.00)	5.98 (0.27)	9.60 (0.56)	
Married	60.21 (0.02)	62.32 (0.71)	52.30 (1.00)	
Never married	12.78 (0.00)	12.22 (0.48)	14.87 (0.68)	
Separated	2.40 (0.00)	2.11 (0.13)	3.50 (0.32)	
Widowed	6.26 (0.00)	6.56 (0.23)	5.14 (0.34)	
PIR				**< 0.001**
≥ 3.5	45.91 (0.02)	49.19 (1.05)	33.61 (1.28)	
0–1	12.03 (0.00)	10.18 (0.43)	18.93 (0.75)	
1–1.35	42.06 (0.01)	40.62 (0.84)	47.47 (1.09)	
BMI				**0.005**
≤ 25	28.64 (0.01)	28.76 (0.54)	28.20 (0.91)	
25–30	33.62 (0.01)	34.18 (0.50)	31.54 (0.79)	
≥ 30	37.73 (0.01)	37.06 (0.62)	40.26 (0.85)	
Education status				**< 0.001**
< high school	15.11 (0.01)	13.97 (0.57)	19.38 (0.87)	
= high school	23.47 (0.01)	22.85 (0.53)	25.78 (0.96)	
> high school	61.43 (0.02)	63.18 (0.87)	54.85 (1.25)	
Hypertension				0.1
No	26.41 (0.01)	26.08 (0.49)	27.64 (0.88)	
Yes	73.59 (0.02)	73.92 (0.49)	72.36 (0.88)	
DM				**0.002**
No	88.95 (0.02)	89.35 (0.32)	87.47 (0.54)	
Yes	11.05 (0.00)	10.65 (0.32)	12.53 (0.54)	
Alcohol user				**< 0.001**
Never	10.60 (0.01)	10.71 (0.47)	10.20 (0.61)	
Former	14.88 (0.01)	14.59 (0.50)	15.99 (0.77)	
Mild	37.83 (0.01)	39.19 (0.73)	32.76 (1.04)	
Moderate	17.26 (0.01)	17.24 (0.42)	17.37 (0.75)	
Heavy	19.42 (0.01)	18.28 (0.49)	23.68 (0.88)	
Smoker				**< 0.001**
Never	52.60 (0.01)	54.60 (0.57)	45.10 (0.98)	
Former	26.73 (0.01)	27.37 (0.47)	24.33 (0.75)	
Current	20.67 (0.01)	18.03 (0.50)	30.57 (0.92)	

BMI, Body Mass Index; DII, Dietary Inflammatory Index; PIR, Poverty Impact Ratio; DM, Diabetes Mellitus; OP, Oral Pain. The bolded numbers represent *p*-values <0.05, which are statistically significant.

### Association between DII and OP

**Figure 2:**
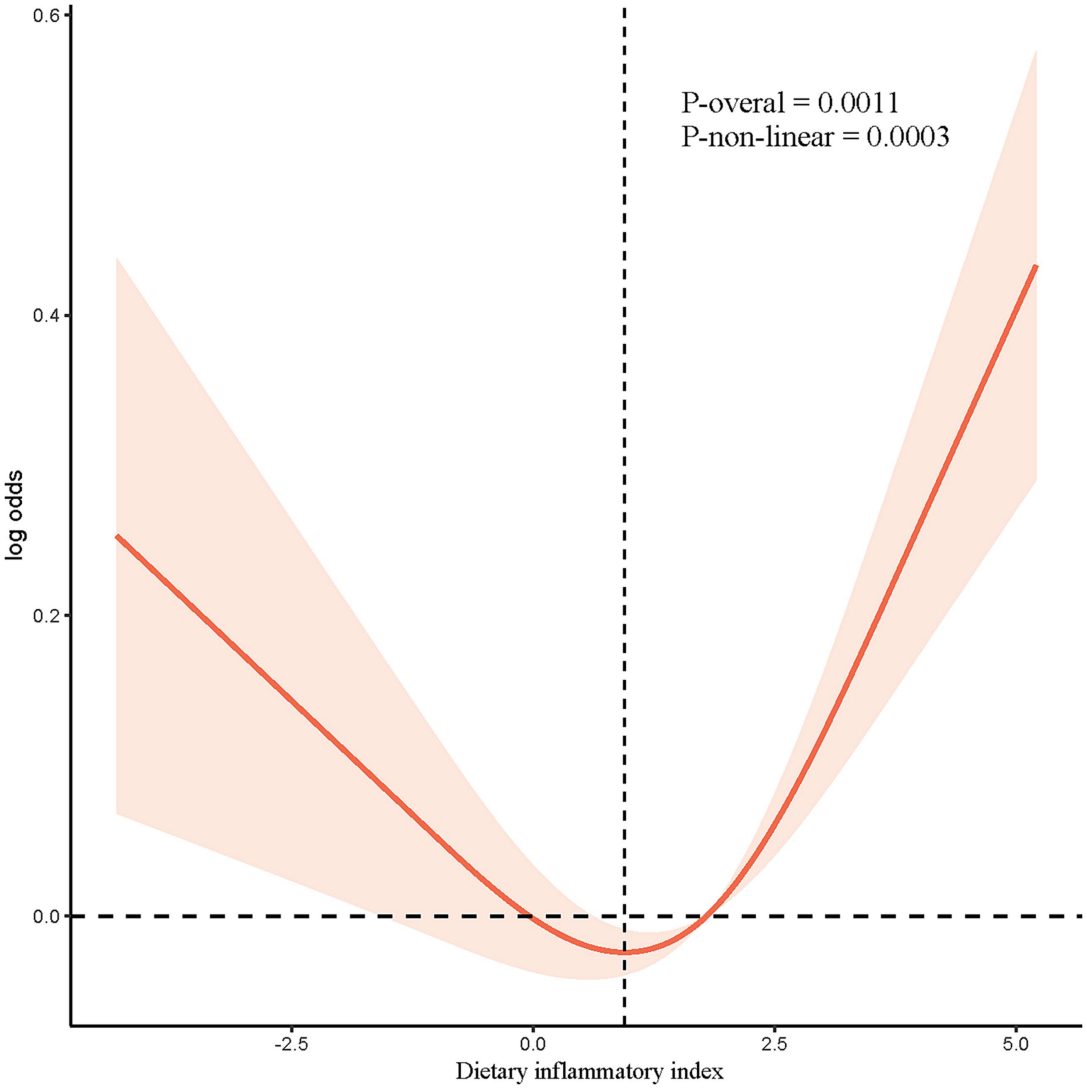
The nonlinear associations between DII and OP. The solid red line represents the smooth curve fit between variables. Blue bands represent the 95% of confidence interval from the fit.

Table 2 shows the association between DII and OP using multiple logistic regression models with and without controlling for covariates. Our results showed a consistently positive association between DII and OP across the unadjusted model 1 (OR = 1.063, 95% CI: 1.040–1.088, *p* < 0.0001), partially adjusted model 2 (OR = 1.058, 95% CI = 1.034–1.083, p < 0.0001), and fully adjusted model 3 (OR = 1.026, 95% CI: 1.001–1.051, *p* = 0.040). Figure 2 shows the restricted spline models based on Model 3, in which the OR was a function of log2. It showed a U-shape association between the DII score and OP (p for non-linearity = 0.0003, p for overall = 0.0011), with a turning point of 0.9479 for the log. Table 3 shows the subgroup analysis, and the results indicated that the association between the DII and OP was consistent across groups. Even though the association was more evident in females, no significant interaction was observed.

**Table 2:** Logistic regression model of dietary inflammatory index and oral pain.

	Model 1	*p* value	Model 2	*p* value	Model 3	*p* value
	OR (95% CI)		OR (95% CI)		OR (95% CI)	
DII	1.063 (1.040, 1.088)	**<0.001**	1.058 (1.034, 1.083)	**<0.001**	1.026 (1.001, 1.051)	**0.040**
Q1	–	–	–	–	–	–
Q2	1.018 (0.900, 1.152)	0.771	1.011 (0.894, 1.144)	0.858	0.962 (0.849, 1.090)	0.542
Q3	1.116 (1.004, 1.241)	**0.042**	1.096 (0.982, 1.223)	0.100	0.991 (0.882, 1.113)	0.874
Q4	1.405 (1.266, 1.560)	**<0.001**	1.376 (1.237, 1.531)	**<0.001**	1.184 (1.060, 1.322)	**0.003**
p for trend		**<0.001**		**<0.001**		**<0.001**

Model 1 without adjustments.

Model 2 additionally adjusted for age (year) and sex (male, female).

Model 3 additionally adjusted for race (Non-Hispanic White, Mexican American, Non-Hispanic Black, Other Race), alcohol user (never, former, mild, moderate, heavy), AST, ALT, BMI (≤25, ≥30, 25–30), smoking status (former, never, current), hyperlipidemia (yes, no), hypertension (yes, no), DM (yes, no). The bolded numbers represent *p*-values <0.05, which are statistically significant.

**Table 3:** Stratified analysis of the association between DII and OP.

Character	OR (95%CI)	*p*-value	*P*-interaction
Age group			0.315
40–59	1.022 (0.975, 1.071)	0.362	
>60	1.000 (0.954, 1.048)	1.000	
20–39	1.033 (0.995, 1.073)	0.085	
Sex			0.097
Male	0.997 (0.964, 1.033)	0.885	
Female	1.040 (1.006, 1.075)	0.020	
BMI			0.877
≥ 30	1.022 (0.984, 1.061)	0.265	
25–30	1.017 (0.969, 1.066)	0.491	
≤ 25	1.013 (0.970, 1.058)	0.546	
Race			0.292
Non-Hispanic White	1.024 (0.987, 1.063)	0.207	
Mexican American	0.955 (0.907, 1.006)	0.082	
Non-Hispanic Black	1.003 (0.967, 1.041)	0.865	
Other Race	1.024 (0.969, 1.082)	0.389	
Hypertension			0.081
No	1.033 (0.999, 1.068)	0.054	
Yes	0.996 (0.963, 1.030)	0.813	
Hyperlipidemia			0.695
No	1.020 (0.975, 1.068)	0.385	
Yes	1.016 (0.988, 1.046)	0.263	
DM			0.709
No	1.017 (0.991, 1.043)	0.204	
Yes	1.028 (0.960, 1.102)	0.421	

Adjusted for the age group, race (Non-Hispanic White, Mexican American, Non-Hispanic Black, other race), BMI group, hypertension, hyperlipidemia, DM.

## Discussion

Our work may be the first study to find a U-shaped association between DII and OP using data from large-scale US adults based on NHANES. In this cross-sectional study based on a US population, we explored the relationship between DII and OP. A positive association was found, which persisted after testing for linear trends and adjusting for demographic data, lifestyle habits, BMI, and common comorbidities. After smoothing the curve, we found that this positive association was U-shaped, with 0.9479 being the significant turning point. Further subgroup analyses indicated no significant differences across different stratifications.

Our study showed that participants’ demographic and clinical characteristics varied by OP status and DII quartile. Participants in the OP group had significantly higher DII scores, indicating a more pro-inflammatory diet. When examining DII in quartiles, we observed a significant trend, with the highest quartile (Q4) showing the greatest risk of OP. This trend was consistent across different age groups and sexes, although the interaction terms were not statistically significant. Our findings are consistent with previous studies showing a positive association between dietary inflammation and OP (21, 29). For instance, one study showed that participants with higher DII scores reported a higher degree of “*Shanghuo*,” which is a traditional concept characterized by “redness, swelling, fever and pain” in Traditional Chinese Medicine (29). Additionally, Luo et al. found that the inflammatory potential of diet, as measured by DII, was associated with an increased risk of oral cancer, suggesting a broader impact of diet on oral health beyond pain alone (21). However, our study is unique in identifying a U-shaped relationship, indicating that both excessively high and low inflammatory diets can increase the risk of OP. This nuance has not been extensively documented in the context of oral health.

The U-shaped relationship between the DII and OP observed in our study might be attributed to several intertwined biological mechanisms. High DII scores, indicative of diets rich in pro-inflammatory foods (e.g., refined carbohydrates, red meats, and sugary beverages), are associated with increased levels of inflammatory markers such as CRP, IL-6, and TNF-*α* (13, 14). These cytokines play crucial roles in inflammation and pain by promoting leukocyte infiltration and macrophage accumulation, which activates pain pathways and exacerbates pain perception (13). Pro-inflammatory diets also elevate oxidative stress by increasing the production of reactive oxygen species (ROS). These ROS can damage cellular structures, including lipids, proteins, and DNA, leading to further inflammation and pain, particularly in sensitive tissues such as the oral mucosa and periodontal tissues (8). High DII diets may influence the gut microbiome composition, promoting the growth of pathogenic bacteria that produce pro-inflammatory metabolites (30). These metabolites can enter systemic circulation and exacerbate inflammation, impacting local oral health through the gut-oral axis (31, 32).

Low DII scores may reflect a deficiency in anti-inflammatory nutrients such as vitamins A, C, D, E, selenium, and omega-3 fatty acids. Diets low in anti-inflammatory nutrients can disrupt hormonal balance, affecting pain perception. Nutritional deficiencies can impair the immune system’s ability to control inflammation effectively (33). Micronutrients can regulate gene transcription factors, such as the proinflammatory nuclear factor kappa B and the anti-inflammatory nuclear factor (erythroid-derived 2)-like 2 (34). A lack of essential vitamins and minerals weakens the body’s antioxidant defenses, increasing oxidative stress and inflammation, thus heightening pain sensitivity (35). For example, omega-3 fatty acids have anti-inflammatory properties that mitigate OP by inhibiting the production of pro-inflammatory eicosanoids (36, 37). Deficiencies in vitamin D and omega-3 fatty acids have been linked to dysregulation of the hypothalamic–pituitary–adrenal (HPA) axis, which is crucial for pain modulation (38–40).

Our findings suggest that maintaining a balanced diet that avoids extremes of the DII spectrum may help manage OP. Clinicians should consider dietary counseling as part of a comprehensive pain management strategy for patients with OP. Emphasizing a diet rich in anti-inflammatory foods while avoiding excessive intake of pro-inflammatory foods could be beneficial. Additionally, our stratified analysis indicates that females might particularly benefit from dietary modifications due to the stronger association between DII and OP in females. Tailoring dietary recommendations to individual patient characteristics, such as sex and specific inflammatory profiles, may enhance the effectiveness of dietary interventions for managing OP.

There are some limitations of this study. First, the present results should be interpreted with caution, as cross-sectional observational studies cannot demonstrate causation and directionality. Second, although confounding factors have been extensively adjusted, there may be other confounders that have not been included and adjusted. Third, Data were based on a self-reported questionnaire, which may be subject to recall bias, particularly concerning OP. Additionally, due to the limitations in the food composition data available in NHANES, we were unable to include plant-based compounds with antioxidant properties in the DII calculation, which may introduce some bias in the results. Future longitudinal study designs with more objective measurement of key variables and controlling for more confounders are needed.

## Conclusion

This study provides evidence supporting the U-shape association between DII and OP, suggesting that maintaining a balanced diet that avoids extremes of the DII spectrum may help manage OP. These findings underscore the importance of dietary interventions in the management and prevention of OP. It is necessary to further study the relationship between DII and OP to determine the specific mechanism of DII on OP.

## Data Availability

The original contributions presented in the study are included in the article/Supplementary material, further inquiries can be directed to the corresponding authors.
