# Supplementary material for: A U-shaped association between dietary inflammatory index and oral pain: a cross-sectional study from NHANES 2003–2018
Source: Front Nutr. 2025 Jul 28;12:1535241. doi: 10.3389/fnut.2025.1535241 (PMC12336255; doi:10.3389/fnut.2025.1535241)
Supplement: Supplementary file 1 [file Data_Sheet_1.docx]

|  |  | **Dietary Inflammatory Index** | | | |  |
| --- | --- | --- | --- | --- | --- | --- |
| **Variables** | **Total** | **Q1** | **Q2** | **Q3** | **Q4** | ***P*-value** |
| Age | 50.02±0.23 | 49.91±0.34 | 50.02±0.29 | 49.97±0.33 | 50.21±0.29 | 0.87 |
| AST | 25.46±0.12 | 26.07±0.26 | 25.56±0.23 | 25.27±0.22 | 24.82±0.34 | **0.02** |
| ALT | 25.64±0.15 | 26.82±0.34 | 26.22±0.33 | 25.33±0.29 | 23.87±0.29 | **< 0.001** |
| Age group |  |  |  |  |  | **< 0.001** |
| >60 | 28.28(0.01) | 26.74(1.02) | 27.61(0.79) | 29.85(0.95) | 29.20(0.82) |  |
| 20-39 | 28.47(0.01) | 28.41(0.89) | 27.28(0.80) | 29.88(0.89) | 28.37(0.90) |  |
| 40-59 | 43.25(0.01) | 44.85(0.90) | 45.11(0.85) | 40.27(0.85) | 42.43(0.99) |  |
| Sex |  |  |  |  |  | **< 0.001** |
| Female | 50.95(0.01) | 37.82(0.76) | 46.33(0.80) | 56.76(0.88) | 65.85(0.69) |  |
| Male | 49.05(0.01) | 62.18(0.76) | 53.67(0.80) | 43.24(0.88) | 34.15(0.69) |  |
| Race |  |  |  |  |  | **< 0.001** |
| Mexican American | 7.43(0.01) | 7.63(0.66) | 8.33(0.69) | 7.14(0.67) | 6.47(0.68) |  |
| Non-Hispanic Black | 9.90(0.01) | 6.73(0.45) | 9.09(0.68) | 10.99(0.81) | 13.51(1.06) |  |
| Non-Hispanic White | 71.87(0.03) | 74.50(1.19) | 71.73(1.27) | 71.21(1.39) | 69.53(1.77) |  |
| Other Race | 10.80(0.00) | 11.14(0.63) | 10.85(0.60) | 10.66(0.65) | 10.49(0.70) |  |
| Marry status |  |  |  |  |  | **< 0.001** |
| Divorced | 11.60(0.00) | 9.60(0.57) | 10.82(0.52) | 12.20(0.59) | 14.28(0.55) |  |
| Living with partner | 6.74(0.00) | 6.92(0.53) | 6.63(0.41) | 6.44(0.51) | 6.97(0.41) |  |
| Married | 60.21(0.02) | 64.83(1.18) | 63.27(0.96) | 58.22(1.03) | 53.29(0.94) |  |
| Never married | 12.78(0.00) | 12.67(0.69) | 11.79(0.63) | 13.03(0.65) | 13.77(0.72) |  |
| Separated | 2.40(0.00) | 1.51(0.18) | 2.19(0.20) | 2.62(0.27) | 3.49(0.29) |  |
| Widowed | 6.26(0.00) | 4.47(0.30) | 5.30(0.30) | 7.49(0.40) | 8.20(0.39) |  |
| PIR |  |  |  |  |  | **< 0.001** |
| ≥ 3.5 | 45.91(0.02) | 56.00(1.42) | 48.94(1.17) | 42.17(1.22) | 34.24(1.23) |  |
| 0 - 1.0 | 12.03(0.00) | 8.87(0.47) | 9.79(0.52) | 12.54(0.67) | 17.85(0.77) |  |
| 1.0 - 3.5 | 42.06(0.01) | 35.13(1.20) | 41.27(1.03) | 45.29(1.07) | 47.92(1.03) |  |
| BMI |  |  |  |  |  | **< 0.001** |
| ≤ 25 | 28.64(0.01) | 32.22(0.90) | 28.65(0.89) | 27.29(0.83) | 25.73(0.75) |  |
| ≥ 30 | 37.73(0.01) | 32.68(0.93) | 36.37(0.81) | 39.62(0.80) | 43.38(0.98) |  |
| 25 - 30 | 33.62(0.01) | 35.09(0.80) | 34.97(0.82) | 33.09(0.71) | 30.90(0.90) |  |
| OP |  |  |  |  |  | **< 0.001** |
| 0 | 78.92(0.02) | 80.74(0.62) | 80.46(0.78) | 78.97(0.64) | 74.89(0.69) |  |
| 1 | 21.08(0.01) | 19.26(0.62) | 19.54(0.78) | 21.03(0.64) | 25.11(0.69) |  |
| Education status |  |  |  |  |  | **< 0.001** |
| < high school | 15.11(0.01) | 10.86(0.62) | 14.36(0.72) | 15.98(0.67) | 20.18(0.88) |  |
| = high school | 23.47(0.01) | 18.86(0.80) | 21.68(0.72) | 25.39(0.79) | 29.01(0.82) |  |
| > high school | 61.43(0.02) | 70.28(1.13) | 63.96(1.05) | 58.63(0.97) | 50.81(1.09) |  |
| Hypertension |  |  |  |  |  | **< 0.001** |
| No | 58.67(0.02) | 61.42(0.89) | 58.93(0.92) | 58.19(0.82) | 55.54(0.84) |  |
| Yes | 41.33(0.01) | 38.58(0.89) | 41.07(0.92) | 41.81(0.82) | 44.46(0.84) |  |
| Hyperlipidemia |  |  |  |  |  | **< 0.001** |
| No | 26.41(0.01) | 30.28(0.84) | 26.39(0.76) | 25.21(0.75) | 23.02(0.73) |  |
| Yes | 73.59(0.02) | 69.72(0.84) | 73.61(0.76) | 74.79(0.75) | 76.98(0.73) |  |
| DM |  |  |  |  |  | **0.001** |
| No | 88.95(0.02) | 90.27(0.45) | 89.09(0.56) | 88.89(0.59) | 87.25(0.55) |  |
| Yes | 11.05(0.00) | 9.73(0.45) | 10.91(0.56) | 11.11(0.59) | 12.75(0.55) |  |
| Alcohol user |  |  |  |  |  | **< 0.001** |
| Never | 10.60(0.01) | 7.91(0.53) | 10.31(0.57) | 10.81(0.62) | 14.00(0.62) |  |
| Former | 14.88(0.01) | 11.74(0.61) | 14.04(0.68) | 15.63(0.62) | 18.85(0.78) |  |
| Mild | 37.83(0.01) | 43.76(1.07) | 39.54(1.01) | 34.98(0.97) | 31.77(0.96) |  |
| Moderate | 17.26(0.01) | 17.41(0.84) | 16.37(0.69) | 18.06(0.66) | 17.23(0.74) |  |
| Heavy | 19.42(0.01) | 19.18(0.86) | 19.74(0.82) | 20.52(0.77) | 18.15(0.62) |  |
| Smoker |  |  |  |  |  | **< 0.001** |
| Former | 26.73(0.01) | 30.03(0.75) | 27.87(0.75) | 25.77(0.83) | 22.47(0.80) |  |
| Never | 52.60(0.01) | 54.89(0.85) | 53.92(0.85) | 50.91(0.96) | 50.14(0.95) |  |
| Current | 20.67(0.01) | 15.08(0.65) | 18.20(0.65) | 23.32(0.78) | 27.40(0.89) |  |

**Table S1** Participants’ baseline clinical and sociodemographic characteristics by DII score quartile

BMI: Body Mass Index; DII: Dietary Inflammatory Index; PIR: Poverty Impact Ratio; DM: Diabetes Mellitus; OP: Oral Pain
