# Supplementary material for: A U-shaped association between dietary inflammatory index and oral pain: a cross-sectional study from NHANES 2003–2018
Source: Front Nutr. 2025 Jul 28;12:1535241. doi: 10.3389/fnut.2025.1535241 (PMC12336255; doi:10.3389/fnut.2025.1535241)
Supplement: Supplementary file 2 [file Data_Sheet_2.docx]

| **Table S2:** The details of DII in NHANES. | | | | | | |
| --- | --- | --- | --- | --- | --- | --- |
| Food parameter | Food parameter in NHANES | code in NHANES(DAY1) | code in NHANES(DAY2) | Overall inflammatory effect score* | Global daily mean intake (units/d)* | sd* |
| Alcohol (g) | Alcohol (gm) | DR1TALCO | DR2TALCO | −0.278 | 13.98 | 3.72 |
| Vitamin B12 (μg) | Vitamin B12 (mcg) | DR1TVB12 | DR2TVB12 | 0.106 | 5.15 | 2.70 |
| Vitamin B6 (mg) | Vitamin B6 (mg) | DR1TVB6 | DR2TVB6 | −0.365 | 1.47 | 0.74 |
| β-Carotene (μg) | Beta-carotene (mcg) | DR1TBCAR | DR2TBCAR | −0.584 | 3718.00 | 1720.00 |
| Caffeine (g) | Caffeine (mg) | DR1TCAFF | DR2TCAFF | −0.110 | 8.05 | 6.67 |
| Carbohydrate (g) | Carbohydrate (gm) | DR1TCARB | DR2TCARB | 0.097 | 272.20 | 40.00 |
| Cholesterol (mg) | Cholesterol (mg) | DR1TCHOL | DR2TCHOL | 0.11 | 279.40 | 51.20 |
| Energy (kcal) | Energy (kcal) | DR1TKCAL | DR2TKCAL | 0.18 | 2056.00 | 338.00 |
| Eugenol (mg) | - | - | - | −0.140 | 0.01 | 0.08 |
| Total fat (g) | Total fat (gm) | DR1TTFAT | DR2TTFAT | 0.298 | 71.40 | 19.40 |
| Fibre (g) | Dietary fiber (gm) | DR1TFIBE | DR2TFIBE | −0.663 | 18.80 | 4.90 |
| Folic acid (μg) | Folic acid (mcg) | DR1TFA | DR2TFA | −0.190 | 273.00 | 70.70 |
| Garlic (g) | - | - | - | −0.412 | 4.35 | 2.90 |
| Ginger (g) | - | - | - | −0.453 | 59.00 | 63.20 |
| Fe (mg) | Iron (mg) | DR1TIRON | DR2TIRON | 0.032 | 13.35 | 3.71 |
| Mg (mg) | Magnesium (mg) | DR1TMAGN | DR2TMAGN | −0.484 | 310.10 | 139.40 |
| MUFA (g) | Total monounsaturated fatty acids (gm) | DR1TMFAT | DR2TMFAT | −0.009 | 27.00 | 6.10 |
| Niacin (mg) | Niacin (mg) | DR1TNIAC | DR2TNIAC | −0.246 | 25.90 | 11.77 |
| n-3 Fatty acids (g) | PFA 20:5 (Eicosapentaenoic) (gm) | DR1TP205 | DR2TP205 | −0.436 | 1.06 | 1.06 |
|  | PFA 22:5 (Docosapentaenoic) (gm) | DR1TP225 | DR2TP225 |  |  |  |
|  | PFA 22:6 (Docosahexaenoic) (gm) | DR1TP226 | DR2TP226 |  |  |  |
| n-6 Fatty acids (g) | PFA 18:2 (Octadecadienoic) (gm) | DR1TP182 | DR2TP182 | −0.159 | 10.80 | 7.50 |
|  | PFA 18:3 (Octadecatrienoic) (gm) | DR1TP183 | DR2TP183 |  |  |  |
|  | PFA 18:4 (Octadecatetraenoic) (gm) | DR1TP184 | DR2TP184 |  |  |  |
|  | PFA 20:4 (Eicosatetraenoic) (gm) | DR1TP204 | DR2TP204 |  |  |  |
| Onion (g) | - | - | - | −0.301 | 35.90 | 18.40 |
| Protein (g) | Protein (gm) | DR1TPROT | DR2TPROT | 0.021 | 79.40 | 13.90 |
| PUFA (g) | Total polyunsaturated fatty acids (gm) | DR1TPFAT | DR2TPFAT | −0.337 | 13.88 | 3.76 |
| Riboflavin (mg) | Riboflavin (Vitamin B2) (mg) | DR1TVB2 | DR2TVB2 | −0.068 | 1.70 | 0.79 |
| Saffron (g) | - | - | - | −0.140 | 0.37 | 1.78 |
| Saturated fat (g) | Total saturated fatty acids (gm) | DR1TSFAT | DR2TSFAT | 0.373 | 28.60 | 8.00 |
| Se (μg) | Selenium (mcg) | DR1TSELE | DR2TSELE | −0.191 | 67.00 | 25.10 |
| Thiamin (mg) | Thiamin (Vitamin B1) (mg) | DR1TVB1 | DR2TVB1 | −0.098 | 1.70 | 0.66 |
| Trans fat (g) | - | - | - | 0.229 | 3.15 | 3.75 |
| Turmeric (mg) | - | - | - | −0.785 | 533.60 | 754.30 |
| Vitamin A (RE) | Vitamin A, RAE (mcg) | DR1TVARA | DR2TVARA | −0.401 | 983.90 | 518.60 |
| Vitamin C (mg) | Vitamin C (mg) | DR1TVC | DR2TVC | −0.424 | 118.20 | 43.46 |
| Vitamin D (μg) | Vitamin D (D2 + D3) (mcg) | DR1TVD | DR2TVD | −0.446 | 6.26 | 2.21 |
| Vitamin E (mg) | Vitamin E as alpha-tocopherol (mg) | DR1TATOC | DR2TATOC | −0.419 | 8.73 | 1.49 |
| Zn (mg) | Zinc (mg) | DR1TZINC | DR2TZINC | −0.313 | 9.84 | 2.19 |
| Green/black tea (g) | - | - | - | −0.536 | 1.69 | 1.53 |
| Flavan-3-ol (mg) | - | - | - | −0.415 | 95.80 | 85.90 |
| Flavones (mg) | - | - | - | −0.616 | 1.55 | 0.07 |
| Flavonols (mg) | - | - | - | −0.467 | 17.70 | 6.79 |
| Flavonones (mg) | - | - | - | −0.250 | 11.70 | 3.82 |
| Anthocyanidins (mg) | - | - | - | −0.131 | 18.05 | 21.14 |
| Isoflavones (mg) | - | - | - | −0.593 | 1.20 | 0.20 |
| Pepper (g) | - | - | - | −0.131 | 10.00 | 7.07 |
| Thyme/oregano (mg) | - | - | - | −0.102 | 0.33 | 0.99 |
| Rosemary (mg) | - | - | - | −0.013 | 1.00 | 15.00 |
| *: The value of overall inflammatory effect score, global daily mean intake (units/d) and sd are derived from references(Shivappa N, Steck SE, Hurley TG, Hussey JR, Hebert JR. Designing and developing a literature-derived, population-based dietary inflammatory index. Public Health Nutr. 2014;17:1689-96.). | | | | | | |
|  |  |  |  |  |  |  |
